# A 5-year retrospective longitudinal study on the incidence and the risk factors of osteonecrosis of the jaws in patients treated with zoledronic acid for bone metastases from solid tumors

**DOI:** 10.4317/medoral.21728

**Published:** 2017-04-08

**Authors:** Maddalena Manfredi, Giovanni Mergoni, Matteo Goldoni, Stefania Salvagni, Elisabetta Merigo, Marco Meleti, Paolo Vescovi

**Affiliations:** 1 Unit of Oral Pathology, Medicine and Laser Surgery, Department of Biomedical, Biotechnological and Translational Sciences (S.Bi.Bi.T), University of Parma, Parma, Italy; 2 Laboratory of Industrial Toxicology, Department of Clinical and Experimental Medicine Parma, University of Parma, Italy; 3 Medical Oncology Unit, University Hospital of Parma, Parma, Italy

## Abstract

**Background:**

The aim of this study was to evaluate the incidence and the risk factors of osteonecrosis of the jaw (ONJ) in a group of patients treated with zoledronic acid (ZA) for bone metastases from solid tumors and enrolled in a preventive dental program.

**Material and Methods:**

This 5-year retrospective longitudinal study included all consecutive oncological patients who underwent at least one infusion with ZA between 2004 and 2011 for bone metastases due to solid neoplasms.

**Results:**

Of the 156 patients enrolled in the study, 17 developed ONJ (10.89%). At the multivariate analysis, severe periodontal disease (*P*=0.025), tooth extraction (*P*<0.0001) and starting the preventive dental program after the beginning of ZA therapy (*P*=0.02) were the only factors which showed a significant association with the occurrence of ONJ.

**Conclusions:**

This study demonstrated the importance of beginning dental prevention before zoledronic acid exposure in reducing ONJ occurrence, especially in the long term. The results of this research show that control of periodontal disease and an increase in the time between tooth extraction and the first ZA administration are recommended in order to reduce the risk of ONJ development.

** Key words:**Osteonecrosis of the jaw, dental prevention, zoledronic acid, incidence.

## Introduction

Bisphosphonate Related Osteonecrosis of the Jaw (BRONJ) is a well-known disease characterized by the presence in the maxillo-facial region of exposed bone for at least 8 weeks in a patient who underwent or who is receiving therapy with bisphosphonates (BP) and who did not undergo radiotherapy of the head and neck (1). This condition may be asymptomatic or can cause swelling of oral and perioral tissues with intense pain, bleeding, persistent purulent discharge and/or draining fistulas, severe halitosis sometimes associated with lower lip paresthesia, mobility and loosening of teeth, causing severe impairment in the quality of live.

The true incidence of BRONJ is still not known (2). A recent systematic review estimated an incidence in oncological patients ranging from 0 to 12,2 per 100,000 patient-years (3).

Intravenous use (IV) of BP is the standard treatment for metastatic disease spread to the bone from solid tumors (i.e. breast, lung, prostate cancer), treatment of multiple myeloma and management of hypercalcemia caused by malignant diseases. IV BP treatment seems to determine a higher risk in the developing the diseases, while the oral use of the drugs longer than 3 years may also increase the risk of BRONJ. In addition, several other systemic and local factors, such as duration of BP therapy, comorbidities, dento-alveolar surgical procedures, infectious dental diseases, concomitant use of drugs such as steroids and anti-angiogenic agents and poor oral hygiene have been reported to be associated with the development of BRONJ (1,4).

A staging system of the condition has been firstly published in 2007 and subsequently modified to obtain a more accurate stratification of patients with BRONJ and specific treatment strategies for each stage (5). At the moment four stages of BRONJ have been identified [0-3] in addition to a “at risk category” indicating asymptomatic patients without clinical evidence of necrotic bone who have been treated with IV or oral BP.

The exact underlying etiopathogenic mechanism of BRONJ is still unclear, therefore preventive measures are restricted to controlling the known risk factors. Several clinical recommendations for operators in the fields of oral health have been proposed, but their real effectiveness in reducing the incidence of BRONJ is unknown because they are mainly based on expert opinion and case series (6).

In the present retrospective study we investigate the 5-years incidence and possible risk factors of BRONJ in a group of patients affected by solid tumor and treated with zoledronic acid (ZA).

## Materials and Methods

The present study was approved by the Ethic Committee of the Azienda Ospedaliero-Universitaria of Parma, Italy. In this retrospective analysis we included all the patients scheduled to receive ZA therapy or already in ZA therapy (4 mg in 250 ml saline solution and infused over 45 min every 28 days) at the Oncological Unit of the Azienda Ospedaliero-Universitaria di Parma for bone metastases due to solid neoplasms and referred to Dentistry Unit, Oral Medicine, Pathology and Laser Assisted Surgery from 01-01-2004 to 01-01-2011 in order to evaluate oral health status and the presence of any possible infection or inflammation which could require dental intervention.

Of the 369 patients initially included, 213 were then excluded from the study because, although they were treated with the ZA, they refused to accept dental care in our unit and were lost to subsequent observations.

For each of the remaining 156 patients demographic data, tumor type, number of ZA administrations, presence of bone metastases, diagnosis of diabetes, medications (corticosteroids, anticoagulants, anticancer therapies) and smoking habits were recorded. We also recorded if the dental care program started before or after the first ZA infusion.

All these patients underwent clinical inspection and an orthopantomography (OPT) examination. In particular patients were checked for periodontal status (tooth mobility, periodontal diseases with particular regard to severe periodontal disease, recorded as loss of clinical attachment >50%, tooth mobility grade II or III, plaque index >50%, bleeding on probing >50%, furcation defects), presence of prosthetic rehabilitation (fixed or removable), presence of root fragments, decay, apical periodontitis and edentulism. All the patients were advised to undergo a dental check every 4 months and a professional oral hygiene recall every 6 to 12 months which included personalized instructions for daily home oral hygiene procedures. Those patients who, during the ZA treatment period, had to undergo tooth extractions followed a specific protocol (Vescovi *et al.*, 2013).

The patients examined before the beginning ZA therapy received specific dental care designed to prevent occurrence of BRONJ. This included extraction of the teeth considered lost to periodontal or carious lesions, treatment of periodontal diseases and 6 to 12-month professional oral hygiene recalls. Patients were not given ZA until 1 month after an extraction. The conservative, endodontic or prosthetic treatments were planned and carried out also in the course of ZA treatment.

The BRONJ staging system approved in 2007 by the AAOMS and subsequently updated in 2009 (1) was used to classified the patients affected by the disease.

- Statistical analysis

We calculated the 5-years incidence of BRONJ in patients who received ZA therapy accordingly with the time they had received dental treatment. The evaluation of the incidence of BRONJ required all patients to be followed for a similar period of time. Consequently, to assess the incidence of BRONJ the period considered started with the date of the first ZA administration and ended with the occurrence of BRONJ or the last clinical examination. For this reason the observation time was standardized for all the patients at 60 months (5 years) from the beginning of ZA treatment.

To assess the possible risk factors of BRONJ we compared the patients affected by BRONJ (BRONJ group) with the patients who did not develop the disease (no-BRONJ group). A multivariate binary model of logistic regression was used including the variables that showed a *P*<0.06 at a first univariate analysis.

The Kaplan Meier test followed by the Log-Rank tests was used to evaluate the difference in time between the onset of BRONJ in patients who started the preventive dental program after the beginning of ZA therapy (group 1) compared to the ones who stared the preventive dental program before the beginning of ZA therapy (group 2).

We evaluated occurrence of BRONJ at 5 years from the first ZA administration in the patients who did not undergo tooth extraction, patients who underwent tooth extraction before ZA therapy and patients who underwent tooth extraction after ZA therapy with Kaplan-Meier analysis and we used Log-Rank tests to assess significance.

Chi-square test was used to compare survival between in BRONJ and no-BRONJ groups.

All the data recorded were analyzed using SPSS (v 21.0, IBM, USA).

Significant level was set at *P*<0.05.

## Results

**Figure 1:**
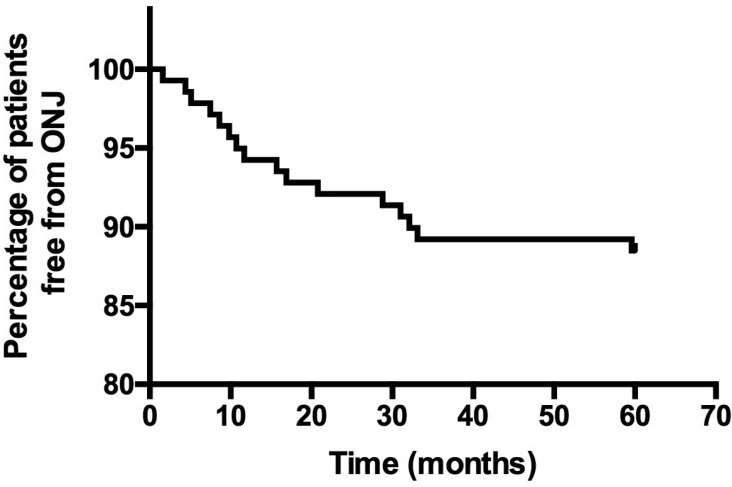
Time of ONJ occurrence since the beginning of ZA therapy.

Out of all the patients enrolled, 17/156 (10.89%) developed BRONJ (group BRONJ: 14 women, 82.4%, 3 men, 17.6%) whereas 139 did not (group no-BRONJ: 104 women, 74.8%, 35 men, 25.2%). Figure 1 shows the Kaplan-Meier estimates of the probability to be free from ONJ. Univariate analysis of possible risk factors between BRONJ group and no-BRONJ is summarized in  Table 1: receiving specific dental prevention after the beginning of ZA therapy (group 1), the presence of severe periodontal disease and tooth extractions were the only variables significantly associated with BRONJ and these factors remained significant after the multivariate analysis ( Table 2).

**Table 1:**
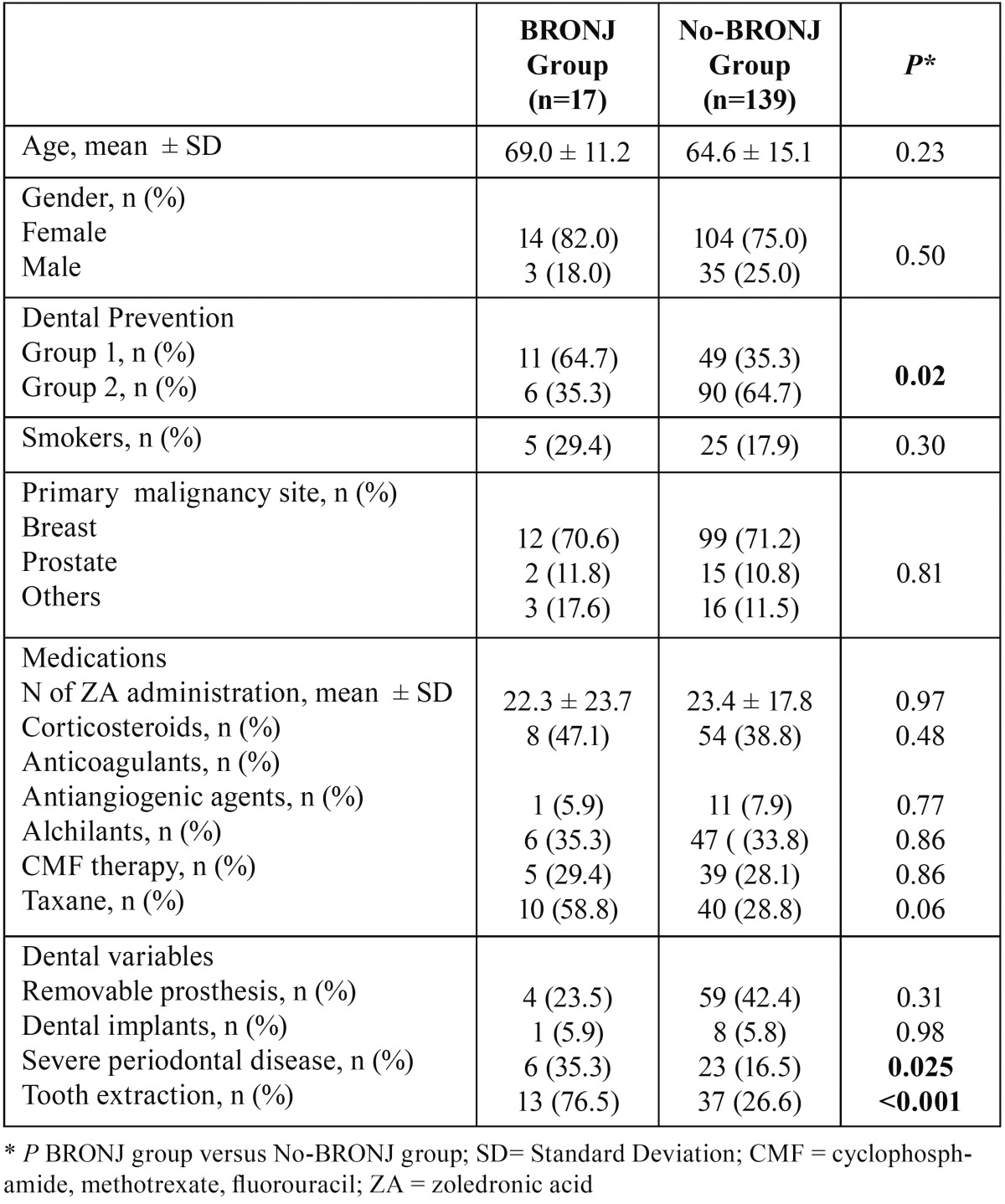
Univariate analysis of BRONJ risk factors.

**Table 2:**
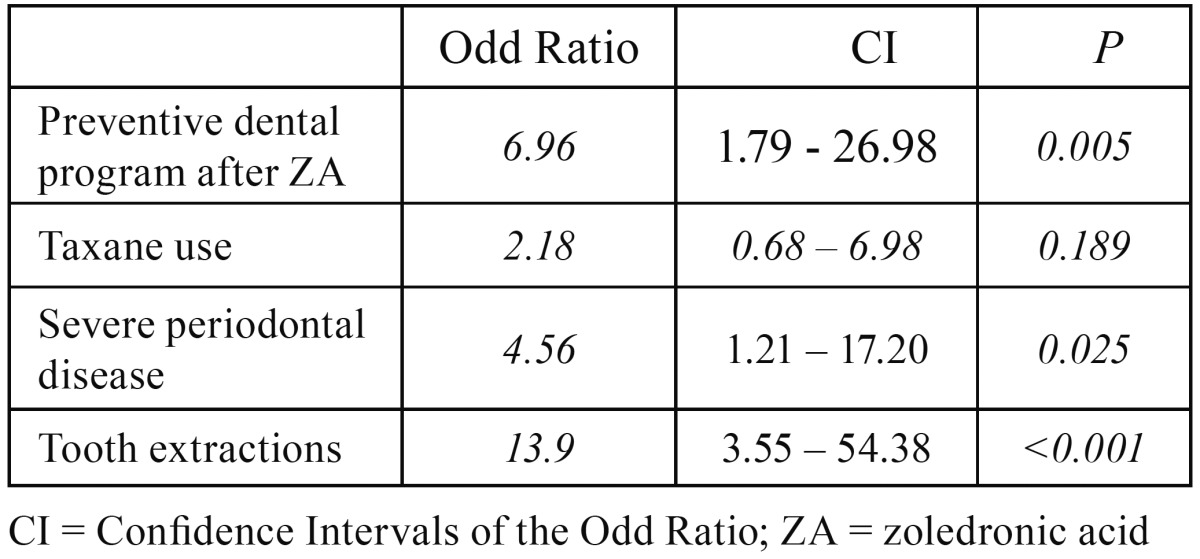
Results of the multivariate logistic regression.

**Figure 2:**
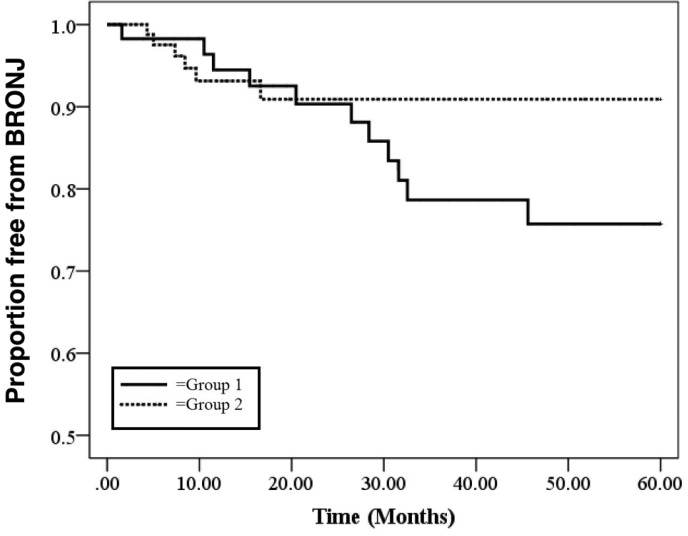
Time of begin of the dental prevention programme and BRONJ. Evaluation of the occurrence of BRONJ at 5 years from the first ZA administration in the group 1 (continuous black line) and group 2 (dotted black line).

Figure 2 shows a Kaplan Meier curve from which it is clear that patients who started the preventive dental program before the beginning of ZA therapy (group 2) tend not to develop BRONJ in the long term in comparison to group 1 patients, who started the preventive dental program after the beginning of ZA therapy. In particular, the final incidence between the two curves is more evident 2 years after the beginning of the ZA therapy.

**Figure 3:**
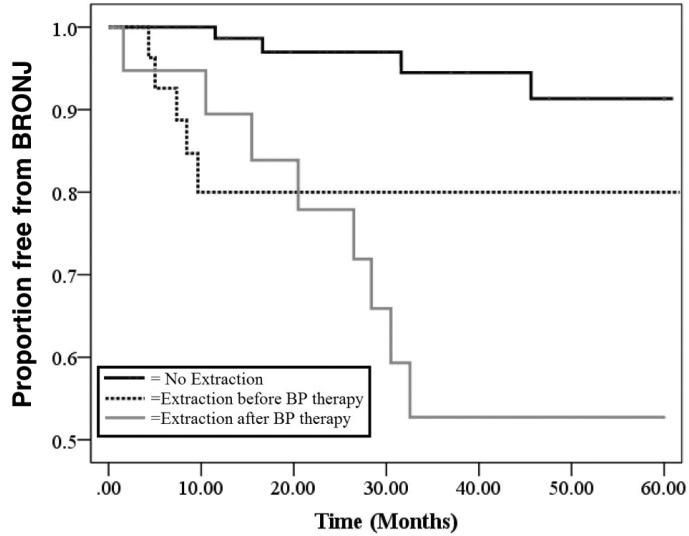
Tooth extractions and BRONJ onset Evaluation of the occurrence of BRONJ at 5 years from the first ZA administration in the patients who did not undergo to tooth extraction (continuous black line), patients who underwent tooth extraction before ZA therapy (dotted black line) and patients who underwent tooth extraction after ZA therapy (gray line).

Figure 3 shows a Kaplan Meier curve of the variable “tooth extractions” compared to patients who had never undergone extractions (n = 106), those who had extractions before ZA therapy (n = 30) and those who had extractions carried out after the beginning of the ZA therapy (n = 20). From this curve it is clear that tooth extractions have an important role in the onset of BRONJ and how the difference is more evident 2 years after the first ZA administration. In particular, comparing patients who had never undergone extractions to those who had extractions before ZA therapy, a statistically significant difference was observed (*P*=0.003). This difference becomes more evident (*P*<0.001) comparing patients who had never undergone tooth extractions to those who had extractions after ZA therapy. No statistical significant difference was obtained comparing patients who underwent tooth extractions before ZA therapy to those who had the extractions after ZA therapy (dotted black line vs grey line, *P*=0.5).

The mandible was the site most affected by BRONJ (9/17, 52.9%), whereas the maxilla was involved in 6/17 cases (35.3%). In two patients (11.8%) BRONJ was present in both jaws. Seven cases were classified as stage I, while 10 as stage II.

The 17 patients who developed BRONJ were treated in our Unit with different protocols:

- 6 patients underwent laser therapy (LT) applications only;

- 4 patients were treated with antibiotics only;

- 3 patients underwent conventional oral surgery and 1 of these received LT applications;

- 4 patients underwent laser-assisted surgery and LT applications.

Complete healing at 6 months (complete healing of the wounds without any symptoms or sign of infection) was obtained for 13/17 patients (76.5%). In particular out of the 13 patients healed, 4 were treated with laser-assisted surgery and LT, 3 with conventional oral surgery, 4 underwent LT only and 2 were treated with antibiotic therapy.

At 5 years 12/17 (70.6%) and 99/139 (71.2%) patients had died in the BRONJ group and in the no-BRONJ group, respectively. At Chi-squared test no statistically significant difference was recorded.

## Discussion

ZA is a potent nitrogen-containing bisposphonate frequently used for the treatment of lytic lesions in patients affected by multiple myeloma and for the management of skeletal-related events associated with bone metastases. In 2007, the U.S. Food and Drug Administration (FDA) also approved the use of an annual infusion of zoledronate for the treatment of postmenopausal osteoporosis (4).

ZA down-regulates osteoclast activities leading to the reduction of bone turnover and inhibition of bone resorption. An uncommon but potential severe side effect of this drug is osteonecrosis of the jaw, firstly reported by Marx et al. in 2003 (7). There is considerable variability in the reported incidence and prevalence of BRONJ occurrence in oncological patients. The reasons of the dishomogeneity in the reported data are several and include use of other drugs that may also impact bone health, such as glucocorticoids or antiangiogenic drugs. Moreover the incidence of ONJ in the oncology patient population may be affected by the type of malignancy being treated. In our study the 5-years incidence was 10.89% and our data confirmed the results of a recent systematic review that estimated an incidence in oncological patients ranging from 0 to 12,2 per 100,000 patient-years (3).

The increasing number of new cases of osteonecrosis associated to ZA and other anti-resorbtive agents lead the scientific community to promote preventive measures in order to reduce the incidence of BRONJ. The effectiveness of preventive strategies is strictly related to the knowledge of the most important risk factors. In our study tooth extraction (*P*<0,0001) and severe periodontal disease (*P*=0,025) were the only dental variables which showed a significant association with the occurrence of BRONJ. Although a minor percentage of BRONJ is related to non-surgical causes, literature clearly demonstrates that BRONJ is preceded by dento-alveolar surgical interventions in the majority of cases (1,4,5). In a multi-center retrospective 4-year study, Vescovi *et al.* found that in 567 cases of BRONJ, 63.8% were associated with a prior dento-alveolar procedure (8). It was calculated that tooth extraction was associated with a 16-fold increased risk for ONJ in cancer patients exposed to zoledronate (9). The role of impaired post extractive healing has been investigated in some studies where tooth extractions were performed in rats treated with BPs (10,11). Analyzing the alveolar socket of BP-treated rats, the authors found delayed bone turn-over, absence of bone resorption, poor vascularity with a low number of osteoclasts and concluded that these findings could be associated with the onset BRONJ.

To better investigate the impact of oral surgery on BRONJ a specific analysis of the timing relating to tooth extractions in relation to BP treatment was performed (Fig. 2). As already reported in literature (1,4,5) the results of this analysis also showed that tooth extractions have a significant impact on the development of BRONJ and that this event is particularly evident 2 years from the beginning of BP treatment.

This study confirms that the incidence of BRONJ in patients who underwent tooth extractions after the ZA therapy is higher in comparison to patients who did not have extractions (*P*<0.001). Moreover, from this analysis, also the patients who had extractions before the beginning of ZA treatment developed BRONJ more frequently (*P*=0.003) with respect to the patients never subjected tooth extraction.

This last finding may reflect the time needed for the bone to recover following the extraction or dental alveolar intervention and the beginning of BP therapy. Although healing events in the tooth extraction socket have been studied extensively in rats, there are few studies that have investigated these processes in humans. Amler and colleagues found that after extraction a blood clot filled the socket (12). After 7 days, the clot was replaced with granulation tissue and then by collagen after 20 days. In this phase bone began forming at the base and the periphery of the extraction socket. At 5 weeks two-thirds of the extraction socket had filled with immature, woven bone. Osteogenic tissue proliferates and trabecular bone is formed followed by a process of bone maturation at 8 weeks from tooth extraction. According to some authors BP could impair this phase of bone maturation, in which osteoclasts are involved, leading to osteonecrosis (13).

In literature there are no data available about the exact time needed from bone to recover from dental extraction and/or surgical procedure in oncological patients. It is clear that individual as well as iatrogenic factors may influence this ability. Usually, based on the experience with patients affected by osteoradionecrosis, several authors suggest that the beginning of BP therapy (or of antioangiogenic treatment) should be delayed until “the extraction site has mucosalized (14-21 days) or until there is adequate osseous healing” (not specified) (4). In our experience and from the analysis of the data obtained in the present study, if the waiting time does not represent a relevant concern for the oncologic progress, we propose that a period longer than 1 month (e.g. 2 months) from the extraction to the first administration of the drug is recommended in order to reduce the risk of BRONJ development.

From our analysis severe periodontal disease was significantly associated with BRONJ onset and this finding is consistent with the literature. A retrospective case control study by Kos showed that the percentage of patients with deep teeth pockets was significantly higher among BRONJ cases (14).

The role of periodontal disease as co-factor in the etiopathogenesis of BRONJ has also been confirmed by two studies on animal models where periodontitis was experimentally induced in rats treated with BP (15-17). The authors found ONJ-like lesions only in rat with induced periodontal disease. According these models, periodontal disease creates periodontal tissue inflammation, which, in normal condition, induces alveolar bone resorption. In presence of bisphosphonates the osteoclasty activity is greatly diminished resulting in retarded resorption of bone that then become exposed to an environment rich in bacterial toxins, inflammatory cytokines and oxidative stress. Such environment is highly toxic to bone cells and results in osteonecrosis.

Therefore, a goal of the preventive management of patient under or scheduled for BP therapy should be the control of periodontal disease, mainly through non-surgical periodontal treatment together with oral hygiene advices.

Regarding the effectiveness of the dental preventive program, we observed a significant reduction in the incidence of BRONJ in patients who received specific dental prevention prior to ZA treatment in comparison to those who were evaluated during ZA treatment (6.3% vs 18.3%).

This data confirms and reinforces the results from other studies in literature, where it has been reported that dental prevention in patients scheduled to start may reduce, but not eliminate, the risk of developing BRONJ in patients treated with ZA (18,19).

As can be noted, in the first 2 years of ZA treatment the incidence of the disease in the two groups is similar. However, this long longitudinal analysis shows that the risk of BRONJ can be reduced up to 3-fold in the long term (after 2 years from the first ZA administration) through a dental prevention program. It could be that other co-factors, both systemic and local, may interfere with the onset of the pathology in the first 2 years of ZA treatment (i.e. genetic factors, underlying diseases, drug therapies).

According to these results, relative to two groups of oncological patients from the same Oncological Unit evaluated during the same timeframe, and in line with the findings of other studies (18,19), dental prevention remains the most significant approach to manage patients’ oral health before and during treatment with BP.
